# Perioperative Anesthesia: Botulinum Toxin Injection in a 6‐Year‐Old Female With “No Burp Disease”

**DOI:** 10.1155/cria/1726887

**Published:** 2026-11-03

**Authors:** Logan D. Applewhite, Lawrence D. Garcia-Reyes, Paul M. Weinberger

**Affiliations:** ^1^ School of Medicine Indiana University Evansville Indiana USA indiana.edu; ^2^ Anesthesia Service Deaconess Health System Evansville Indiana USA deaconess.com; ^3^ Deaconess Health System Evansville Indiana USA deaconess.com

**Keywords:** “botulinum toxin”, “case report”, “pediatric anesthesia”, “perioperative anesthesia”, “retrograde cricopharyngeus dysfunction”

## Abstract

Retrograde cricopharyngeus dysfunction is a chronic condition involving inability of the cricopharyngeal muscle to relax in a task‐specific manner (eructation). Symptoms commonly include bloating, significant discomfort, excessive flatulence, and often self‐imposed limitations in oral diet and activity. Here, we report a 6‐year‐old patient with several years of symptoms who underwent surgical injection of botulinum toxin under general anesthesia. Anesthesia with propofol and sevoflurane provided adequate sedation and analgesia. Nitrous oxide was avoided due to theoretical concern for impaired ability to clear esophageal and abdominal gas which can lead to postoperative nausea, vomiting, and aspiration risk.

## 1. Introduction

Retrograde cricopharyngeus dysfunction (RCPD), also referred to as “abelchia” or “no‐burp disease,” is a task‐specific dysfunction of the cricopharyngeal muscle that prevents relaxation to release esophageal air (that would normally trigger eructation). In RCPD patients, the cricopharyngeal muscle relaxes appropriately when swallowing but fails to relax in response to distension caused by esophageal air [1]. Far from being a minor annoyance, RCPD patients suffer from four key symptoms: an inability to belch, abdominal fullness/bloating, audible gurgling noises in the chest/lower neck, and excessive flatulence. Another common but not universal symptom is an impaired emesis mechanism, in which vomiting is difficult or painful, but not impossible. This leads to many patients developing emetophobia. Often these lead to social inhibition/isolation, alterations in diet (e.g., avoidance of carbonated beverages), and development of compensatory behaviors such as “air vomiting” where patients purposefully trigger their gag reflex. While other pharyngeal, esophageal, and gastrointestinal pathologies should be considered, syndromic presentation and the inability to belch is considered sufficient for clinical diagnosis and treatment referral. Positive response to botulinum cricopharyngeal injection is both therapeutic and diagnostic, and other ancillary approaches are discouraged due to the accuracy of patient self‐diagnosis [2]. Botulinum toxin (onabotulinumtoxin A) inhibits the release of acetylcholine at the neuromuscular junction, leading to localized muscle paralysis. In the case of RCPD treatment, botulinum toxin is injected into the cricopharyngeal muscle, thus inducing relaxation and allowing air to pass *retrograde* through the upper esophageal sphincter. Patients are usually able to eructate voluntarily within 1 week of injection, and interestingly this treatment effect appears to frequently outlast the 3‐4‐month direct botulinum toxin paralysis on the cricopharyngeal muscle [3].

RCPD was first noted in medical literature in 1987; however, botulinum toxin injection as a treatment was first brought forward in 2019. It remains underrecognized, with many patients going through extensive work‐up before being referred for botulinum toxin therapy [1]. Botulinum injection can be performed in the office or in the operating room, with many laryngologists preferring the operating room with sedation or under anesthesia [4]. The potential anesthetic implications of RCPD are undefined and deserve further consideration. This manuscript adheres to the applicable EQUATOR guidelines, and written Health Insurance Portability and Accountability Act consent for the publication of this paper was obtained from the patient’s legal guardian.

## 2. Case Description

This patient was a six‐year‐old 19.1 kg female who presented for botulinum toxin injection therapy via rigid esophagoscopy under general anesthesia for treatment of RCPD. The patient’s parents first noticed unexplained periodic pain and abdominal discomfort around the age of three. Her symptoms were initially attributed to recurrent acute otitis media infections, however subsequent tympanostomy tube placement and adenoidectomy failed to impact the intermittent pain. Dietary modification was largely ineffective, and celiac disease workup was negative. Multiple imaging studies including abdominal plain‐film radiography (Figure 1), small bowel barium enema fluoroscopy (Figure 2), and upper gastrointestinal esophageal fluoroscopy demonstrated increased air in the gastrointestinal system, but no structural abnormalities. Her stomach would be flat during the morning prior to eating and would progress toward impressive distention by the end of the day due to air trapping. The patient’s symptoms also significantly worsened with activities that increased mouth breathing, such as swimming. During these episodes, the patient would experience abdominal bloating, inability to belch, and subsequent excessive flatulence, but never had observed reflux or emesis.

**FIGURE 1:**
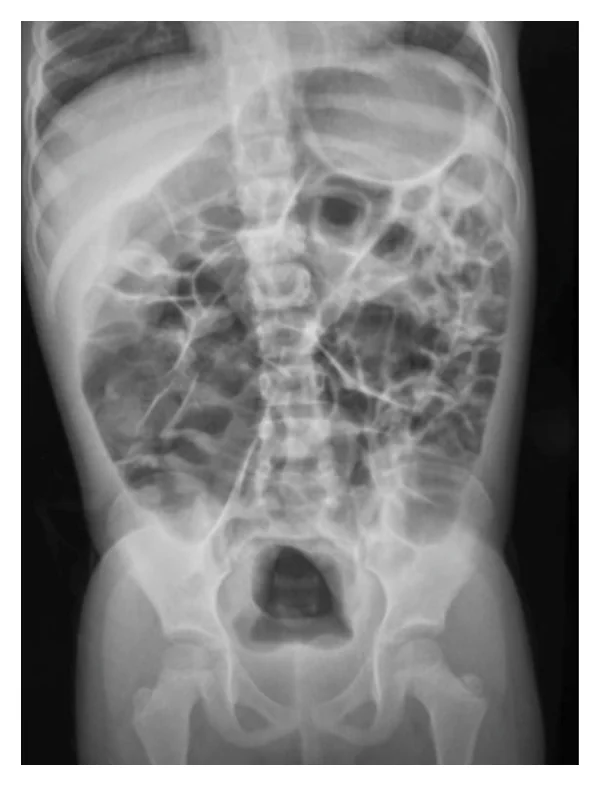
Abdominal kidney, urine, and bladder X‐ray prior to botulinum toxin injection.

**FIGURE 2:**
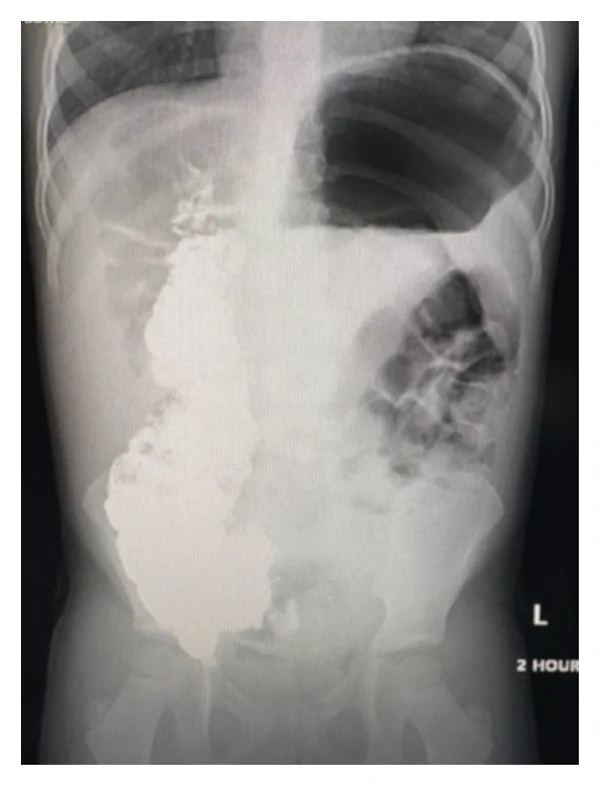
Small bowel with barium enema X‐ray prior to botulinum toxin injection.

After personal online research, the parents identified RCPD as a possibility. The patient was referred to an Ear, Nose, and Throat surgeon with a laryngology fellowship. The surgeon agreed that her symptoms were in congruence with RCPD, and that it was reasonable to proceed with treatment. As such, an endoscopic botulinum toxin injection under general anesthesia was scheduled.

### 2.1. Preoperative Evaluation

NPO status was verified. Allergies were reviewed. There were no medical contraindications to anesthesia. Her Mallampati Score was one and her ASA class was I. Discussion of anesthetic agents identified a theoretical risk for esophageal and abdominal distension if nitrous oxide (a common induction adjunct at this facility) were used in this patient due to its known propensity for diffusion and accumulation in enclosed spaces such as the stomach, thorax, and middle ear space. In this patient with inability to vent esophageal gas, this could increase risk for postoperative nausea and vomiting.

### 2.2. Anesthesia

Upon arrival in the operating room, the patient was placed in the supine position. She was initially administered 7% sevoflurane and 93% O_2_ via face mask at a flow rate of 8 L per minute. This allowed peripheral intravenous access to be obtained and administration of 30 mg propofol bolus to provide general anesthesia. A cuffed size six endotracheal tube was placed with a McGrath MAC blade size 2 video laryngoscope. The airway was secured in one attempt and was secured at an insertion depth of 18 cm. Placement was verified with auscultation and capnometry. 8 mg dexamethasone and 2 mg ondansetron were administered. Anesthesia was maintained with 2.9% sevoflurane at 2 L per minute, then 2.2% sevoflurane at 2 L per minute, then weaned at 0.4% sevoflurane at 8 L per minute until extubation. The patient’s SpO_2_ was 100% throughout the case, EtCO_2_ averaged 41 mmHg. 100 mL of Lactated Ringer were administered over the course of the case. The total time under anesthesia was 21 minutes.

### 2.3. Intraoperative Events

After general anesthesia induction, a preoperative time‐out was performed. A Dedo rigid scope was advanced into the oropharynx and then advanced to the postcricoid region to expose the upper esophageal sphincter. The patient was then placed into Lewy suspension. Epinephrine‐soaked pledgets were placed for a vasoconstrictive field effect. Under endoscopic guidance, 20 units of botulinum toxin were injected submucosally into the cricopharyngeal muscle, staggered across the exposed portion of the cricopharyngeal muscle in three injections. This dosing was selected using a one unit/kg calculation. The toxin was diluted to a concentration of 5 units/0.1 mL (50 units/mL), and 0.4 mL was injected in total. After injection, the scope was removed, and the patient was weaned from anesthesia.

### 2.4. Postoperative Course

The patient recovered appropriately in PACU. She was alert, awake, and oriented with stable vital signs on room air. There was no evidence of anesthetic complications or recall. The patient tolerated the procedure well and had adequate analgesia. She did not experience any postoperative adverse effects from surgery.

### 2.5. Follow‐Up

Several days following surgery, the patient began to belch (for the first time in her life). Initially, belches were of limited volume, but within two to 3 weeks, her belches became more robust. At her most recent outpatient follow‐up 12 months status postinjection, the patient reported significant symptom relief, and her parents are pleased with the surgical outcome.

## 3. Discussion

This patient fit the classic presentation of RCPD. She had an inability to belch, painful abdominal bloating, gurgling noises in her chest and lower neck, and excessive flatulence [2]. She was also never observed vomiting, even during periods of extreme distention, indicating likely emesis mechanism impairment, emetephobia, or a combination of both. In addition, this patient was self‐referred, a common trend seen in previously documented RCPD cases.

Botulinum toxin injection typically results in muscle paralysis for three to 4 months. It is effective at treating RCPD with around a 90% initial response rate that typically lasts for 3‐4 months. 4%–45% of patients have recurrence of symptoms at 6 months, but repeat injections have a higher long‐term success rate. The mechanism for this response pattern is currently unknown [5]. As of 12 months status postinjection, this patient has had no recurrence of symptoms.

Anesthetic induction of pediatric surgical procedures frequently uses nitrous oxide; however, this was avoided for several reasons. Addition of nitrous oxide to general anesthesia increases postoperative nausea and vomiting [6, 7]. This patient had risk factors for postoperative nausea including young age, and female sex. Nitrous oxide is also associated with a two‐fold risk increase of intraoperative bowel distension [8]. In this patient with pre‐existing increased abdominal air and impaired ability to clear abdominal air, the use of nitrous oxide carried the possibility of additional abdominal distension.

RCPD is likely underrecognized rather than truly rare and can have a profound impact on patient quality of life. Surgical injection of botulinum toxin under general anesthesia allowed this patient to belch for the first time in her life and has resulted in resolution of abdominal distension and related symptoms. Anesthesia with propofol and sevoflurane provided adequate sedation and analgesia with no side effects or complications. Nitrous oxide was avoided in this patient due to theoretical concern for postoperative nausea, vomiting, and abdominal bloating.

## Funding

No funding was received for this manuscript.

## Conflicts of Interest

The authors declare no conflicts of interest.

## Data Availability

The data that support the findings of this study are available on request from the corresponding author. The data are not publicly available due to privacy or ethical restrictions.
